# Valuation Impacts of Environmental Protection Taxes and Regulatory Costs in Heavy-Polluting Industries

**DOI:** 10.3390/ijerph17062070

**Published:** 2020-03-20

**Authors:** Wen-Jun Tu, Xiao-Guang Yue, Wei Liu, M. James C. Crabbe

**Affiliations:** 1 Business School, Ningbo University, Ningbo 315211, China; 2 Department of Computer Science and Engineering, School of Sciences, European University Cyprus, 1516 Nicosia, Cyprus; xgyue@foxmail.com; 3 CIICESI-ESTG, Porto Polytechnic, 4610-156 Felgueiras, Portugal; 4 Business School, Qingdao University, Qingdao 266100, China; wei.liu2@sydney.edu.au; 5 Wolfson College, Oxford University, Oxford OX2 6UD, UK; james.crabbe@wolfson.ox.ac.uk; 6 Institute of Biomedical and Environmental Science and Technology, University of Bedfordshire, Luton LU1 3JU, UK; 7 School of Life Science, Shanxi University, Taiyuan 030006, China

**Keywords:** environmental protection tax, market investor response, regulatory costs, environmental disclosure, regional legal institutions, China

## Abstract

In 2016, the issue of the Environmental Protection Tax Law indicated the enhancement of environmental protection in China. This study examines the market reaction to firms in heavy-polluting industries, and the effects of external legal institutional quality and internal environmental disclosure on firm value around the passage of Environmental Protection Tax Law. Using an event study approach coupled with ordinary least square regressions, the researchers find a significantly negative market reaction to firms in heavy-polluting industries, but this negative reaction varies depending on the expected increase in future regulatory costs. Specifically, the above negative reaction is stronger when the firm reveals that itself or its subsidiary belongs to heavy-polluting industry, however it would be mitigated when a firm is in a region with better quality of legal institutions or discloses environmental improvement activities. Overall, the results are consistent with the market perceiving that the environmental protection tax law enacted would increase regulatory costs for firms in heavy-polluting industries, and also show the higher-quality regional legal institutions and more efforts on environmental protection could relieve the market’s pessimism caused by uncertainty.

## 1. Introduction

Public and government attention to the environment are increasing along with the surge in pollution levels in China since 2013 [1,2]. In order to mitigate environmental and ecological problems and make China’s pollution under control, the Environmental Protection Tax Law was enacted on December 25, 2016 at the 25th Session of the Standing Committee of the Twelfth National People’s Congress and has been implemented since January 1, 2018. The shift from the pollutant discharge fee to environmental protection tax probably increases the regulatory costs (e.g., discharge costs and costs to deal with environmental issues), defined as “costs incurred by a firm in response to or as a result of proposed or enacted government regulations” [3]. 

The literature reveals the effects of regulatory costs on firm value based on predicting the government’s future environmental regulations after environmental disasters or increasing haze levels [1,3]. Logically, since environmental accidents or increasing environmental risks, legislative reforms would be implemented to create an institutional environmental mandate to encourage business players to develop sustainably. Blacconiere and Patten confirmed the negative intra-industry reaction and the positive role of extensive environmental disclosures in firms’ financial reports on the firm value, based on US chemical firms’ samples [3]. Li et al. reported that Chinese firms providing corporate social responsibility (CSR) reports and in high polluting industries were relatively shielded from higher regulatory costs through exploring the market valuation of expected regulatory costs [1]. However, due to the uncertainty of future regulatory policies, the literature shows the positive effects of CSR reports but inconsistent reactions for several polluting industries. Based on a certain regulatory policy, He et al. found out the most sectors in China experience negative market reactions to the passage and implementation of Environmental Protection Tax Law, and the state ownership influences the market reactions [4]. However, the literature has not differentiated the market reactions on the passage of the law and the implementation of the law, and fails to understand the influence of the new tax on the market reactions conditional on firms’ pollution level and environmental information disclosure which may heavily influence the market’s prediction for firms’ future regulatory cost. In the context of the Chinese transitional economy, this study mainly investigates the market’s perception of future regulatory costs around the passage of the new environmental policy. This study also examines how this perception varies across firms in industries with different pollution levels, in different regions and with distinct environmental disclosures. The passage of the Environmental Protection Tax Law, as an external policy, provides us a good opportunity to understand the effects of regulatory costs on firm value through examining the market reaction to firms at the time the law was passed. In this study, the authors argue that the stock market would respond more negatively to firms in heavy-polluting industries since the investors perceive the increase of regulatory costs heavy-polluting firms faced after the shift from pollutant discharge fees to environmental taxes. In line with this argument, based on heavy-polluting firms, the authors further argue that the market’s perception for the expected increase in regulatory costs would vary depending on regional legal institutions and whether firms embrace environmental information disclosure.

The regional legal quality represents the effectiveness of regulation implementation [5]. The authors expect that firms located in a region with higher-quality legal institutions are less influenced by this environmental regulation change. Those firms are viewed as highly compliant players, since the implementation of discharge fees and other pollutant control regulations should be relatively rigid in a region with better legal institutions. In addition to legal institutions, the growing concern with environmental sustainability results in the increasing requirement of environmental information disclosure [6,7], which is of particular interest to regulators, investors and stakeholders, especially in emerging markets [8]. The authors argue that firms revealing their heavy-polluting status or disclosing environmental information are more likely to draw the market’s concern with the increase of regulatory costs because of the current mandatory environmental reporting requirements in China. However, the authors also argue that disclosures regarding environmental improvement activities are expected to moderate the market’s concern with the increase in future regulatory costs.

Using an event study approach combined with ordinary least square regressions, the authors find a significant, overall negative market reaction to firms in heavy-polluting industries at the time the tax law passed, and this negative reaction varies depended on regional legal quality and firms’ disclosures of their heavy-polluting status and environmental improvement activities. Specifically, the negative market reaction was stronger when the firm admitted that itself or its subsidiary belonged to heavy-polluting industry. However, it could be mitigated by higher-quality regional legal institutions or the disclosure of environmental improvement activities particularly by a narrative language.

This study seeks to make three main contributions. First, the authors explore market perceptions of regulatory costs in a unique setting, where the regulations are under transitions and the regional legal institutions are developed unevenly. Second, in the aspect of regulatory costs, this study enriches the research on how macro-level policies influence firm value through examining the market reaction to the passage of the Environmental Protection Tax Law. Third, our research enhances the understanding of the role of environmental information disclosure on firm value. The inconclusive arguments about the role of environmental information disclosure in previous literature is partly because of the potential endogeneity problems [6]. It is often difficult to determine which direction is right, “doing well by doing good” or “doing good while doing well”, since good environmental responsibility and firm value may support each other. In this case, distinct firm value change came from the variance of expected regulatory costs for different firms around the law passed. The passage of the Environmental Protection Law, as an exogenous event, allows us to explore the influence of environmental protection activities on firm value without the endogeneity problem.

The outline of this paper is organized as follows: the second section introduces the institutional background in China, followed by the development of research hypotheses based on relevant literature. After that, this paper outlines our sample and empirical methodology with the analyses of empirical results. The final section makes the conclusions.

## 2. Environmental Protection in China

Due to the rapid economic development through industrialization and urbanization, China has become the world’s second-largest economy since 2010 and the biggest energy-consuming country in the world [9]. Along with the economic development, environmental pollution is becoming a serious problem in China, especially air and water pollution. The surge of air pollution increases the days of heavy pollution and puts one third of Chinese cities struggling with haze issues [10], while the severe water pollution causes the problem of drinking water scarcity for millions of Chinese citizens [2,11].

In order to mitigate severe environmental problems, the Chinese government has enacted a series of laws and regulations to handle environmental problems. On the one hand, in order to control emissions, a set of environment-related laws has been issued in China, such as the Environmental Protection Tax Law in 2016 by the National People’s Congress [12]. The environmental protection tax as the first Pigouvian tax in China is used to avert and mitigate negative externalities [13]. Based on the rule of Pigouvian taxes, this new tax is expected to reduce pollution by charging a price rather than imposing restrictions directly on the amount of pollution emission. The newly passed environmental protection tax in general follows the previous system of pollutant discharge fees but differentiates itself from the fee in the following three aspects. First, the taxes collected are retained at the local level instead of allocating 10 percent of the fee to the central government as usual. Second, the environmental protection tax applies different tax rates depending on the level of pollution rather than use a uniform rate for all polluters. Third, the violating cost for failure to pay is also enhanced by the law, i.e., the upper bound for the fine increasing from threefold the amount of tax liability unpaid to fivefold.

In addition, the Chinese government has enacted a set of regulations to enforce and encourage firms to disclose environmental information. In 2003, *the Bulletin on Disclosure of Corporate Environmental Information* announced by *the State Administration of Environmental Protection* (SAEP, renamed as *the Ministry of Ecology and Environmental* in 2018) required firms which discharge pollutants above the levels specified by governments to disclose environmental-related information annually [12]. In 2008, *the Regulation on Environmental Information Disclosure for Listed Firms* issued by *the Shanghai Stock Exchange* enforced corporate environmental information disclosure for listed firms when itself or its subsidiary belongs to heavy-polluting industries recognized by the government, and allowed voluntary disclosure of environmental information for the rest of firms. *The Guidelines on Environmental Information Disclosure for Listed Firms* issued by SAEP in 2010 not only enhanced the implementation of previous regulation, but also required that firms in the selected industrial sectors go through environmental audits and make their results public if they intend to apply for public listing or refinancing in the stock market. However, those official rules only provided general guidance rather than detailed ones to generate variation in transparency and breadth of environmental information disclosures by firms located in different regions due to the variation in law enforcement by local authorities [14].

## 3. Hypotheses 

### 3.1. The Market Reaction to Firms in Heavy-Polluting Industries

Existing literature has investigated whether the change in expected regulatory costs triggers abnormal market reaction for firms in certain industries. Bowen, Castanias, and Daley [15] and Hill and Schneeweis [16] reported the negative effect of the Three Mile Island nuclear accident on the shareholder value of firms in the electric utility industry, while Blacconiere and Patten found a significant overall negative market reaction for firms in the chemical industry after Union Carbide’s chemical leak in Bhopal [3]. Based on the increasingly severe haze events in China, Li et al. found an overall negative market reaction at the time of the haze event [1]. However, they indicate firms in heavy-polluting industries are relatively shielded from higher regulatory costs evidenced by the same market response to haze events for firms in polluting and non-polluting industries.

Different from Li et al.’s focus on the uncertain future regulatory costs related to haze, the change in regulatory costs caused by the passage of the Environmental Protection Tax Law is relatively easy to document [1]. The increased regulatory costs vary across firms with different pollution levels. Firms in heavy-polluting industries suffer from higher pollutant emission costs after tax implementation. On the one hand, the change in the allocation mechanism by implementing the environmental protection tax allows local authorities to keep more fiscal income after the new law was enacted. On the other hand, the application of different tax rates depending on the level of pollutant emission and the increased violating cost should increase pollutant emission costs for firms to discharge pollutants, in particular heavy-polluting firms. Hence the newly implemented tax law provides local authorities more pressures as well as incentives to monitor local firms’ environmental violations and may increase regulatory costs of firms discharging pollutants. In addition, managers in heavy-polluting firms are most likely to carry out environmental activities after the law was passed, which will also increase the regulatory costs. Existing literature suggests environmental activities may increase firm value through improving a firm’s reputation. However, since the existence of conflicts of interests between managers and shareholders [17], few environmental activities result in a value increase for firms. The shareholders hardly know how those environmental activities work due to the information asymmetry. Managers may use those activities to fulfill their own demands such as building reputation or an environmentally friendly image of themselves rather than shareholders’ profits [18].

Therefore, we propose the following hypothesis:

**Hypothesis** **1.** *The market reaction to the passage of Environmental Protection Tax Law is negative for firms in heavy-polluting industries.*

### 3.2. The Effect of Regional Legal Quality

Although China has made significant efforts in establishing a legal system to control pollution, the enforcement of environmental laws remains a problem [19]. In addition, China’s administrative decentralization implemented in the last century decentralizes the control of central government onto local enterprises and government institutions, which partly results in the uneven development of regional legal quality [20,21]. The legal institutions, in particular the effectiveness of law enforcement, varies across regions in China [22,23].

This regional variation in environmental law enforcement is noted in previous literature [24,25], which finds considerable regional variations in monitoring and punishments of environmental destruction [24]. Those variations are mainly rooted in the effectiveness of local government management [25]. 

Prior to the Environmental Protection Tax, the local Environmental Protection Bureau (EPBs) monitored and measured the level of pollutants and was responsible for collecting discharge fees. The local governments directly managed the local EPBs, and sometimes tended to prioritize economic growth over environmental quality [26,27]. Under low-quality legal institutions, the local authority’s power was relatively overwhelmed, and the judicial system was less effective [26]. Specifically, local EPBs suffered from more serious internal management problems and risk-averse problems (i.e., being afraid to upset local regulated actors) [28]. The weaker the legal institutions were, the more serious local environmental law enforcement was undermined, so that previously the tax compliance was relatively low with low-quality legal institutions [29].

After this reform, local EPBs are only responsible to monitor and evaluate pollutant emissions, and local Taxation Bureaus guided by both local government and the higher Taxation Bureau are in charge of environmental tax collection. The change in responsible institutions improves the compliance cost for firms, particularly for firms with worse tax compliance history. Hence, the market is likely to respond more negatively to firms located in regions with lower-quality legal institutions due to the relatively larger increase in expected regulatory costs for those firms. We propose the following hypothesis:

**Hypothesis** **2.** *Firms in heavy-polluting industries located in a region with higher-quality legal institutions generates a less negative market reaction around the passage of the Environmental Protection Tax Law.*

### 3.3. The Effect of The Heavy-Polluting Status Disclosure

According to disclosure requirements by the Shenzhen and Shanghai Stock Exchanges, a firm must state whether it or its subsidiary belongs to heavy-polluting industries in annual financial report and disclose its environmental information if the firm is in the category of heavy-polluting industries. However, not all firms expected in heavy-polluting industries reveal their heavy-polluting status as required. For instance, CSG Holding Co. Ltd. belongs to non-metallic mineral product manufacturing and construction industries which were recognized as heavy-polluting industries in the 2008 and 2010 Guidelines for Environmental Information Disclosure for Listed Firms. However, in its 2015 financial report, the firm stated that it did not belong to heavy-polluting industries. Similarly, many firms should be classified in heavy-polluting industries but had not truly declared their polluting status in financial reports.

Unlike a developed capital market dominated by institutional investors, the majority of Chinese investors are individuals with less experience and investment skills [30,31]. Chinese investors might heavily relay on the superficial information revealed by listed firms rather than deep analysis based on complex sourced information. Hence, given the idiosyncrasy of the Chinese stock market, we propose the following hypothesis:

**Hypothesis** **3.** *Firms in heavy-polluting industries disclosing its heavy-polluting status in annual reports generate a more negative market reaction around the passage of the Environmental Protection Tax Law.*

### 3.4. The Effect of Environmental Information Disclosure

The effect of environmental information disclosure on firm performance is, to date, still inconclusive. Based on the stakeholder theory, one strand of literature suggests that environmental information disclosure will enhance corporate environmental image and reduce the market’s prediction of future regulatory costs [3,32]. On the other hand, the competing view based on shareholder theory indicates environmental protection improvement activities entail costs so that those activities have not created value or have even hampered the firm value [33,34].

The value of environmental information for investors is to make sure firms would not violate regulations and suffer from high violation costs. The environmental information disclosure may reduce the market’s uncertainty about the firm’s future illegal activities. Specifically, the disclosure of detailed environmental improvement activities would reduce investors’ uncertainty about the firm’s increasing tax burden and make them believe firms still experience sustainable development without the influence of increasing environmental protection tax. 

Based on previous studies [35,36], this study explores two types of environmental improvement information disclosure: symbolic environmental improvement information using a narrative language and substantive environmental improvement information using quantitative data.

Symbolic environmental improvement information enhances a firm’s social legitimacy [36]. Organizational legitimacy is vital for a firm’s survival and success under conditions of complexity [37]. Recently, harmonious, stable and prosperous development has gradually replaced the sole economic goals along with improving living conditions, and many new regulations and laws relating to environmental protection have been enacted [38]. The institutional change is caused by shifts in the assessment of the legitimacy by a society [39]. As a “symbolic means of inducing cooperation”, the narrative disclosure about how to improve the ability of environmental protection and pollutant emission control, is one of the means to secure a firm to enhance its legitimacy among stakeholders under institutional change [40]. Apart from symbolic environmental improvement information, substantive environmental improvement information reduces information asymmetry between the market and a firm [30]. The negative market reaction results from investors’ perception of the increase in future regulatory costs after the new taxation law was implemented. However, the substantive environmental improvement information by quantitative data can mitigate the market’s concern about the increasing emission costs. From the perspectives of legitimacy and information asymmetry, we propose the following hypotheses:

**Hypothesis** **4a.** *Firms in heavy-polluting industries experience a less negative market reaction around the passage of the Environmental Protection Tax Law if they reveal environmental improvement activities using a narrative language.*

**Hypothesis** **4b.** *Firms in heavy-polluting industries experience a less negative market reaction around the passage of the Environmental Protection Tax Law if they reveal environmental improvement activities using quantitative data.*

## 4. Data and Methodology

### 4.1. Data Source

The researchers combine an event study approach with an ordinary least square regression to document the market reaction to the passage of the Environmental Protection Tax Law on December 25th, 2016 and the effect of expected regulatory costs on firm value. The researchers retrieved data from two sources. The stock trading data and firm-level governance and financial information for A-share listed firms were collected from the Chinese Stock Market & Accounting Research (CSMAR) database [41]. CSMAR, jointly produced by the Hong Kong Polytechnic University and Shenzhen GTI Financial Information Limited, is widely used in studies relating to the Chinese context [42,43]. The researchers hand collected the data about a firm’s heavy-polluting status and other environmental information from financial, CSR and sustainability reports. Data on the quality of regional legal institutions was obtained from the National Economic Research Institute’s Provincial Index of Marketization (NERI), created and updated by Fan, Wang and Zhu [44].

### 4.2. Sample Selection

In order to examine whether the market responds differently to firms in heavy-polluting and non-heavy-polluting industries, our initial sample consisted of all Chinese A-share firms listed in Shanghai and Shenzhen stock exchanges on December 25, 2016 after excluding firms without qualified stock trading data to implement event study. The legislative day (i.e., December 25, 2016) is chosen rather than the exercise day (i.e., January 1, 2018) because the former day is the formal announcement day for the enforcement of the Environmental Protection Tax Law which could capture the majority of the effect on stock prices [45]. Compared with the legislative day, the exercise day may contain little or no new information because the enforcement of the new tax law has been certain already.

The researchers selected the sample according to following criteria: (1) excluding firms without enough stock prices information for event study and (2) excluding the special treatment (ST) listed firms.

Further, the researchers differentiate heavy-polluting firms from non-heavy-polluting firms. The selection of heavy-polluting firms is based on research by Du et al. [14,46]. According to the most updated classification of heavy-polluting industries in the Guidelines on Environmental Information Disclosure for Listed Firms issued by SAEP in 2010, the researchers defined sixteen heavy-polluting industries (the sixteen heavy-polluting industries cover thermal power, steel, cement, electrolytic aluminium, coal, metallurgy, chemical, petrochemical, building materials, papermaking, brewing, pharmaceuticals, fermentation, textiles, leather and mining). Following the Guidelines for the Industry Classification of Listed Companies (2012 Revision) issued by China Securities Regulatory Commission (CSRC), the researchers matched defined heavy polluting industries with three-digit industry codes issued by CSRC. Hence, listed firms with following industry codes are included in the sample of heavy-polluting firms: mining industries (B06 for coal, B07 for petroleum and natural gas, B08 for ferrous metal, B09 for non-ferrous metal, B10 for non-metallic mineral), manufacturing industries (C15 for alcoholic drink and beverage, C17 for textiles, C19 for leather, C22 for papermaking, C25 for coking and nuclear fuel processing, C26 for chemical raw materials and chemical products, C27 for pharmaceuticals, C28 for chemical fiber, C30 for non-metallic mineral products, C31 for ferrous metal smelting and calendaring, C32 for non-ferrous metal smelting and calendaring, C33 for metal products), and the thermal production and supply industry (D44). 

After sifting through the data, the number of observations used for event study to calculate the short-term stock-price performance of firms was 2619, including 721 firms in heavy-polluting industries and 1898 firms in non-heavy-polluting industries. 

Since only firms with it or its subsidiary belonging to heavy-polluting industries are enforced to disclose environmental-related information in reports, this study further explores the effects of regional institutions and environmental information disclosures on firm value within heavy-polluting firms. Hence the researchers excluded observations (i.e., firms) in non-heavy-polluting industries and those with missing data for variables included in regression models. The final sample for cross-sectional analysis consisted of 650 firms (i.e., observations) in heavy-polluting industries.

### 4.3. Estimation Period and Market Reaction

The researchers used event study to determine whether and how the market responds differently to firms in heavy-polluting industries and non-heavy-polluting industries by calculating the short-term stock-price performance of firms (i.e., the cumulative abnormal return, CAR) around the passage of the tax law. The CAR around the passage of the tax law is calculated based on a market model.

The researchers first calculated normal return of the stock for each firm by estimating the following standard market model:

(1) $$R_{it}=\alpha_{i}+\beta_{i}R_{mt}+\varepsilon_{it},$$

where, $R_{it}$ is the daily rate of return on stock *i* at time *t*, $R_{mt}$ is the equal-weighted daily market return on Shanghai and Shenzhen markets at time *t*, $\alpha_{i\;}and\;\beta_{i}\;$are parameters with the latter representing the systematic risk of stock *i*, $\varepsilon_{it}$ is the random error term, with E($\varepsilon_{it}$) =0, t is the day measured relative to the event with t=0. Day 0 should represent the time of the Environmental Protection Tax Law passed, but the law was passed on Sunday and day 0 has no trading information. Therefore, the researchers redefined day 0 as the next day after the law passed. A 200-day estimation period from t = −210 to t = −11 relative to day 0 (where t=0) was applied to estimate parameters. The researchers computed the abnormal return of the stock *i* for a given day using $AR_{it}$ as shown below:

(2) $$AR_{it}=R_{it}-({\hat{\alpha }}_{i}+{\hat{\beta }}_{i}R_{mt}),$$

where ${\hat{\alpha }}_{i}$ and $\hat{\beta }$i are estimates from Equation (1) for firm *i*. The CAR for each stock *i*, was calculated by summing up the abnormal returns over the event window in accordance with the following formula:

(3) $${CAR}_{i}=\sum_{i=m}^{n}AR_{it},$$

where (m, n) represents the event window. The crucial part in the event-study method is the length of the event window used. This study utilizes a wide range of event windows (−5, −1; −5, 0; −4, 0; −3, 0; −2, −0; −1, 0; −5, 1; −5, 2) to examine the market reaction.

### 4.4. Model

Based on the sample of firms in heavy-polluting industries, the paper uses cross-sectional analysis to investigate the effects of external legal institutions and internal environmental information disclosure on firm value in the aspect of the market’s perception of expected future regulatory costs. The researchers use Ordinary Least Square regressions (OLS) and adjust standard errors for heteroscedasticity by applying the Huber-White sandwich estimators [47,48]. Our main regression model is specified as follows:

(4) $$CARs=\beta_{0}+\beta_{1}Regional\;legal\;quality+\beta_{2}Heavy-polluting\;status+\beta_{3}Environmental\;information\;disclosure+\beta_{4}Controls+\varepsilon ,$$

In order to control the potential effects of outliers, the researchers winsorized all firm-level continuous variables at the 1st and 99th percentiles. Additionally, to mitigate endogenous problems, all firm-level independent and control variables were lagged by one year. Moreover, given that the market reactions might vary across regions and industries, industry fixed effect and region fixed effect were controlled in this study. Specially, industry effect dummy variables were designed based on two-digit industry codes of firms, while region effect dummy variables were designed based on seven different regions in China (i.e., Huanan, Huadong, Huazhong, Huabei, Xinan, Xibei, and Dongbei).

### 4.5. Variables Measurement

#### 4.5.1. Dependent Variables

The researchers calculated a series of CAR within various event windows, and chose CAR (−5, 0) as our dependent variable in main regressions to carry out cross-sectional analysis following the principle that the event window should be short enough to enhance the power of the analysis and sufficiently long to incorporate the full effect of the event [49]. The Environmental Protection Tax Law was passed on the last day of the 25^th^ Session of the Standing Committee of the Twelfth National People’s Congress of the People’s Republic of China held during December 19 to 25, 2016. Since the conference schedule was made public on the first day of the meeting, December 19 (day −5) and the first market trading day December 26 (day 0) after the passage of the law were chosen as the beginning and the end of the event window respectively. 

#### 4.5.2. Independent Variables

To examine the effects of regional legal quality, we used *Rlegal* measured by the legal environmental index in 2015 from NERI indices to represent the quality of regional legal institutions. For the disclosure of firm’s heavy-polluting status, we used *Heavy-pollutingF*, a dummy variable, to measure how a firm in heavy-polluting industries discloses its heavy-polluting status. The variable equals one if a firm discloses itself or its subsidiary belongs to heavy-polluting industries in annual reports, and zero otherwise.

In addition, the researchers tested the effects of the disclosure of environmental improvement activities in the following ways. Yin et al. classified environmental information disclosure into symbolic-style disclosure indicating a firm’s environmental strategy and goals by a narrative language, and substantive-style disclosure reporting quantitative data [36]. Following the definition of Yin et al., this research continued to look at whether the symbolic and substantive disclosure of environmental improvement activities mitigate the market’s concern with the increasing regulatory costs for the firm. Symbolic disclosure means the disclosure using subjective information (i.e., textual descriptions and nonquantitative information), such as ”reduce pollution emission through upgrading machine and improving producing process”, “take activities to pursue the low carbon economic development ”, “develop clean energies”, “closely pay attention to the change in the regulations about environment protection and timely improve the firm’s standards”, “focus on green innovation to pursue sustainable development”, etc. Substantive disclosure means the disclosure using objective information (i.e., the accurate quantitative data). Hence, two dummy variables *EimprovementA* and *EimprovementC* were incorporated in regressions. *EimprovementA* equals one if a firm describes environmental improvement activities by a narrative language, and zero otherwise. *EimprovementC* is equal to one if a firm reported an accurate amount of environmental improvement cost, and zero otherwise.

#### 4.5.3. Control Variables

Based on prior CSR and environmental information disclosure (EDI) studies [12,28], we controlled for the following two sets of variables in cross-sectional regressions: (1) firm-level basic characteristics: *Fsize*, measured with the logarithmic transformation of the total asset; *Fage*, measured with the number of years since a firm’s initial public offering (IPO) as the end of 2016; Tobin’s Q, measured with market value of the firm divided by the total asset, which indicates growth opportunities for a firm; *ROE*, return on equity, measured as net income divided by the total equity; *Dratio* (the debit ratio), measured with the ratio of the total liability to the total asset, reflecting the influence of resource constraints. (2) corporate governance characteristics: *Econcentration*, measured with the ownership percentage of the ultimate controlling shareholder reflecting the influence of the controlling shareholder; *Idirector*, measured with the ratio of the number of independent directors over the total number of the board of directors to reflect the quality of corporate governance; *SOE*, a dummy variable, equaling to one if the ultimate controlling shareholder of a firm is a government agency or government controlled state-owned enterprises, and zero otherwise. The industry and region fixed effects were controlled in this study as well.

## 5. Results and Discussions

### 5.1. Sample Statistics and Correlation

Table 1 presents our sample distribution for event study based on the provinces where the listed firms are registered. All provinces in mainland China are included in this study. In the sample, Guangdong has the largest number of listed firms, but Zhejiang has the largest number of listed heavy-polluting firms.

Table 2 presents the descriptive statistics and Pearson correlations of the variables used in cross-sectional analysis. The correlations among variables were relatively low, and the largest and mean value of Variance Inflation Factor (VIF) scores for all regressions (reported in the last two lines of Table 4) were far from the cut-off point of 10 indicated by Kutner, Nachtsheim, and Neter, which alleviate concerns for multicollinearity [50]. In addition, CAR (−5, 0) was generally significantly related to most control variables (at the 5% level or better), providing assurance that the controls identified based on prior research are appropriate in explaining the shareholder value. Moreover, *Heavy-polluting firms* was negatively and significantly related to the market reaction while *Rlegal* and *EimprovementA* had a positive and significant effect, suggesting that firms disclosing its heavy-polluting status have inferior market reaction but firms located in regions with higher-quality legal institutions or reporting detailed improvement activities enjoy superior market reaction. Multivariate regressions in the next subsections are used to examine whether these preliminary results continue to hold after accounting for firm-level characteristics.

### 5.2. The Overall Market Reaction

Table 3 presents the average daily abnormal returns (ARRs) and cumulative abnormal returns (CARs) around the passage of the Environmental Protection Tax Law in terms of polluting character for different event windows in Panels A and B respectively. In addition, we draw t 1 based on Panel A to visually show the differences in ARRs and CARs for firms in heavy-polluting industries and non-heavy-polluting industries.

**Figure 1:**
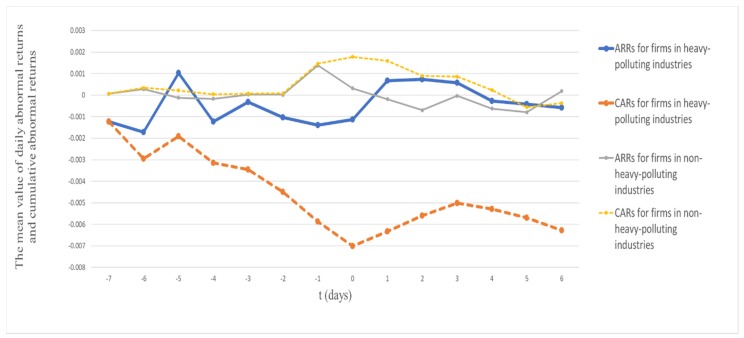
The average daily abnormal returns (ARRs) and cumulative abnormal returns (CARs) around the passage of the Environmental Protection Tax Law in terms of industry character. (a) The vertical axis is the mean value of ARRs and CARs along with the horizontal timeline t (days), day 0 is the first trading day after the passage of the law since the law was passed on Sunday. (b) The thick solid and dashed curves represent the mean value of ARRs and CARs along with t (days) for firms in heavy-polluting industries respectively, while the thin solid and dashed curves represent the mean value of ARRs and CARs along with t (days) for firms in non-heavy-polluting industries respectively.

Panel A of Table 3 and Figure 1 show generally negative AARs for firms in heavy-polluting industries from seven days before (t = −7) to one day after the passage of the law (t = 0), and continuously declining CARs for those firms during the day −5 to 0. Panel B reports CARs for subsample firms for a range of event windows. The mean value of CARs of firms in heavy-polluting industries were negative for all event windows and statistically significant for most event windows at the 10% level or above, but the mean values of CARs of firms in non-heavy-polluting industries were generally positive but insignificant around the law passed. In addition, we applied a t-test for the differences in means of CARs between firms in heavy-polluting industries and non-heavy-polluting industries. The results show the differences between two groups were significantly different from zero based on two-tail p-values for most of the event windows [51]. Specifically, based on a one-tail p-value, the means of CARs for firms in heavy-polluting industries were significantly lower than the means of CARs for firms in non-heavy-polluting industries. All results in Table 3 and Figure 1 support our Hypothesis 1, which indicates that the market responded much worse to firms in heavy-polluting industries at the time the Environmental Protection Tax Law passed. Our findings are consistent with previous research reporting an overall significantly negative intra-industry market reaction to negative environmental events announcements for firms in certain industries [3]. Our results further explain He et al. (2019)’s finding that market reactions to Environmental Protection Tax Law vary across sectors [4]. Specifically, our findings also provide evidence of the significant influence of increasing environmental regulatory costs on the value of firm in heavy-polluting industries, which opposes Li et al. (2017)’s argument that firms in heavy-polluting industries would be relatively shielded from higher regulatory costs [1]. 

Since only firms in heavy-polluting industries are required to report environmental information, this study only include heavy-polluting firms in the following cross-sectional analysis by OLS regressions. Specifically, given the leakage of the information, the researchers used CAR (−5, 0) as the dependent variable in main regressions and test the sensitivity of our findings by using CAR (−5, −1), CAR (−5, 1) and CAR (−5, 2) in the robustness check.

### 5.3. Cross-Sectional Results

Table 4 presents cross-sectional results based on the sample of firms in heavy-polluting industries. Model 1 shows the coefficient estimates of the baseline regression model including only the control variables. 

The coefficients on *Fage*, *ROE*, *Dratio* and *Econcentration* were consistently negative and significant at the 5% or 10% level, indicating that the investors responded negatively to firms with a shorter listing period, higher prior performance, more debts and more concentrated ownership structure. The market expected that firms with the above characteristics may suffer from a larger increase in future regulatory costs after the new tax law was enacted, consistent with previous studies. Specifically, the market was concerned the younger firms engaging more in environmental conservation [14], the higher ROE of firms resulted from illegally reducing tax burden [52], and firms with more concentrated ownership structure had more incentive to avoid pollutant discharge fees illegally due to the entrenchment effect [53].

Model 2 reports the effect of regional legal quality. The coefficient estimate for *Rlegal* was significantly positive at the 1% level, providing a strong support to our Hypothesis 2 that the market has more confidence in heavy-polluting firms located in a province with higher-quality legal institutions. The explanation may be that the rigorous enforcement of pollutant discharge fees policy and other environmental laws in those provinces leads to the higher environmental law compliance for local firms, so that the market predicts the less increase in future regulatory costs for firms located in regions with better legal institutions after the tax law was enacted.

Model 3 shows whether a firm disclosing its heavy-polluting status influenced the market reaction. The coefficient estimate of *Heavy-pollutingF* was significantly negative at the 1% level, strongly supporting our Hypothesis 3 that firms disclosing its heavy-polluting status triggered a worse market reaction around the new tax law passed because the market predicts those firms would bear a higher increase in tax burden due to their heavy-polluting character.

Model 4 provides an analysis pertaining whether and how the disclosure of environmental improvement information influenced the firm value. The coefficient on *EimprovementA* was significantly positive at the 1% level, which supports Hypothesis 4a that the detailed symbolic description for environmental improvement would lead to superior market reaction. Consistent with our prediction about the influence of substantive disclosure, the coefficient on *EimprovmentC* was positive. However, the coefficient was not significant, only partially supporting our Hypotheses 4b that the disclosure of accurate environmental improvement investment would lead to a better market reaction. Our results further provide evidence for Yin et al. (2019)’s argument that the symbolic-style disclosure using a narrative language contributed more to the firm’s profitability than the substantive-style disclosure using quantitative data.

Model 5 includes all variables examined in this study and shows that our results were consistent in the final model. All hypotheses and results are summarized in Table 5. 

### 5.4. Robustness Check

Although the independent and control variables are lagged by one year and industry and region fixed effects are controlled, the researchers used a Hausman test to further ensure there were no endogeneity problems for independent variables chosen in this study. Based on the results of Hausman tests, all p-values are not significant even at the 10% level so that the endogeneity would not be a serious problem for this study. The results are available in Panel A of Table 6.

Additionally, the researchers conducted robustness tests to verify whether our main results still hold by using CARs within different event windows. In Panel B of Table 6, we used CAR (−5, −1), CAR (−5, 1) and CAR (−5, 2) to replicate main analyses and find our main results mostly remain unchanged. All control variables in the main regressions are included but not reported here to conserve space. The full appendix displaying the results of the robustness check is available upon request.

## 6. Conclusions

As environmental quality becomes ever-more important for the public, the Chinese government implements increasingly strict environmental protection regulations. However, there are few studies to look at how environmental policy change influences firm value. This study examines the value effects from the passage of the Environmental Protection Tax Law on heavy-polluting firms as well as firm- and regional-level influential factors in the aspect of expected future regulatory costs. Consistent with published literature, this study also finds an overall significantly negative market reaction to heavy-polluting firms at the time of the tax law passed and provides evidence on the firm value change resulted from the passage of policies and regulations expected to influence future regulatory costs [1,3].

Given the unique nature of Chinese institutions, this study further investigated whether external regional legal institutions and internal environmental information disclosure explain the variation in the market value change of heavy-polluting firms caused by the passage of the Environmental Protection Tax Law. Consistent with our predictions depending on the market’s perception of the increase in future regulatory costs, the market reaction was more negative for firms disclosing their heavy-polluting status or disclosing environmental information without detailed improvement activities, but less negative for firms located in regions with higher-quality legal institutions or disclosing detailed environmental improvement information.

Considering the mandatory environmental information disclosure requirements in China, the market may have viewed firms revealing their heavy-polluting status or disclosing environmental information in reports as likely to experience a larger increase in regulatory costs to comply with the Environmental Protection Tax Law [54]. However, reflecting the value of stronger legal institutions, more favorable reactions occurred for heavy-polluting firms located in regions with higher-quality legal institutions, in which local firms have greater compliance with law and may be less influenced by the enforcement of increasingly stringent environmental regulations. In addition, the environmental improvement information, in particular symbolic disclosure using a narrative language helps to relieve the market’s concern with increasing future regulatory costs. Consistent with the Environmental Protection Tax Law (i.e., the first green tax law) in China focused on discouraging and reducing environmental pollution [55], it appears that the market finds stronger regional legal institutions and prior environmental improvement activities to be valuable through reducing the uncertainty of future regulatory costs in the era of sustainable development.

This study has several implications. First, the findings that expected regulatory costs related to the passage of the Environmental Protection Tax Law can affect the shareholder value in China suggest that market investors should pay attention to social development trends and policy changes so that they can adjust their investment to follow general trends. For instance, they could switch to more environmentally-friendly industries and avoid the uncertainty stemming from the increasingly stringent pollution control by the government. Second, the positive influence of the firm disclosing its environmental protection by subjective description on the firm value suggest that market investors do care about firms’ environmental protection value and strategies described by a narrative language. Therefore, a firm should mitigate the market’s concern with the future regulatory costs resulted from the increasingly stricter environmental regulations by disclosing detailed environmental information, in particular narrative information about environmental law compliance and environmental improvement.

The implications of our research should be considered within the confines of the limitations. First, this study only takes firms in heavy-polluting industries as the sample to investigate the role of external regional legal quality and internal environmental information disclosure on the firm value around the passage of Environmental Protection Tax Law. The reason is there are lack of environmental disclosure data for most firms in non-heavy-polluting industries who are not required to report environmental information compulsorily. In view of this, future research can collect environmental information data for non-heavy-polluting firms to understand the influence of voluntary disclosure on firm value. Second, this study does not apply carbon emissions data to group heavy and non-heavy polluting firms due to the missing of verified carbon emissions data and the punishment coverage of environmental protection tax. Further studies can use carbon emissions data at firm level to extend our understanding of the influence of regulatory costs on firm value. 

In closing, the findings are consistent with the notion that the perception of future regulatory costs will influence the market reaction to environmental events or the enforcement of environmental policies. Moreover, this research also shows the external regional legal institutions and internal environmental informal disclosure could influence the market’s perception of the increase in future regulatory costs around the passage of new laws.

## Figures and Tables

**Table 1:** Sample distribution by provinces for event study.

Provinces	Firms in Non-Heavy-Polluting Industries	Firms in Heavy-Polluting Industries
Anhui	53	25
Beijing	201	44
Fujian	71	21
Gansu	14	11
Guangdong	339	63
Guangxi	21	11
Guizhou	8	10
Hainan	16	9
Hebei	25	22
Henan	37	30
Heilongjiang	24	9
Hubei	61	19
Hunan	51	24
Jilin	19	13
Jiangsu	193	71
Jiangxi	17	17
Liaoning	44	19
Inner Mongolia	6	17
Ningxia	4	7
Qinghai	5	5
Shandong	95	57
Shanxi	10	24
Shaanxi	30	9
Shanghai	178	34
Sichuan	66	28
Tianjin	31	8
Tibet	5	7
Xinjiang	27	11
Yunnan	10	15
Zhejiang	212	68
Chongqing	25	13
	1898	721

**Table 2:** Descriptive statistics and Pearson correlation matrix for variables in main regressions.

Variables	A	B	C	D	E	F	G	H	I	J	K	L	M
**Plane A: Descriptive statistics**													
Observation	650	650	650	650	650	650	650	650	650	650	650	650	650
Mean	−0.006	22.431	12.483	3.343	0.044	0.439	35.9	0.373	0.463	8.25	0.32	0.298	0.332
Sd	0.042	1.315	6.601	2.76	0.297	0.21	17.228	0.053	0.499	4.631	0.467	0.458	0.471
Min	−0.208	18.924	1	0.871	−2.205	0.031	0.3	0.25	0	1.33	0	0	0
Max	0.134	28.504	25	28.338	5.713	0.994	89.41	0.6	1	16.19	1	1	1
**Panel B: Pearson correlation matrix**													
A: CAR (−5, 0)	1												
B: Fsize	−0.016	1											
C: Fage	−0.111 *	0.203 *	1										
D: Tobin’s Q	−0.022	−0.598 *	−0.157 *	1									
E: ROE	−0.095 *	−0.073 *	−0.048 *	0.204 *	1								
F: Dratio	−0.093 *	0.417 *	0.301 *	−0.282 *	−0.214 *	1							
G: Econcertation	−0.084 *	0.371 *	0.025	−0.165 *	−0.032 *	0.061 *	1						
H: Idirector	−0.037 *	−0.032 *	0.031 *	0.094 *	−0.022	0.011	−0.025	1					
I: SOE	−0.037 *	0.345 *	0.446 *	−0.303 *	−0.098 *	0.326 *	0.339 *	−0.047 *	1				
J: Rlegal	0.133 *	0.004	−0.199 *	0.001	0.140 *	−0.194 *	0.009	−0.075 *	−0.179 *	1			
K: Heavy-pollutingF	−0.159 *	0.266 *	0.119 *	−0.167 *	−0.069 *	0.174 *	0.129 *	−0.057 *	0.170 *	−0.036 *	1		
L: EimprovementA	0.140 *	0.341 *	0.162 *	−0.222 *	−0.021	0.079 *	0.121 *	−0.014	0.230 *	0.050 *	0.165 *	1	
M: EimprovementC	0.0113	0.111 *	0.085 *	−0.157 *	−0.124 *	0.186 *	−0.014	−0.047 *	0.085 *	−0.144 *	0.132 *	0.025	1

**Notes:** The table reports the basic descriptive statistics and correlation for dependent, independent and control variables in the regressions. The sample size for cross-sectional analysis was 650. Mean and Sd refer to the mean and standard deviation of each variable. *P<0.05.

**Table 3:** Average abnormal returns and cumulative abnormal returns.

**Panel A: Average Abnormal Returns (AARs)**								
**Event days**	**Whole sample**		**Firms in heavy-polluting industries**		**Firms in non-heavy-polluting industries**			
	ARRs	CARs	ARRs	CARs	ARRs	CARs		
−7	−0.00029	−0.00029	−0.00123	−0.00123	0.00007	0.00007		
−6	−0.00028	−0.00057	−0.00172	−0.00295	0.00027	0.00034		
−5	0.00019	−0.00037	0.00103	−0.00192	−0.00012	0.00022		
−4	−0.00046	−0.00083	−0.00122	−0.00314	−0.00017	0.00004		
−3	−0.00007	−0.00090	−0.00031	−0.00346	0.00003	0.00007		
−2	−0.00027	−0.00117	−0.00103	−0.00449	0.00001	0.00008		
−1	0.00062	−0.00056	−0.00139	−0.00587	0.00138	0.00146		
0	−0.00008	−0.00064	−0.00113	−0.00701	0.00032	0.00178		
1	0.00005	−0.00059	0.00068	−0.00633	−0.00019	0.00160		
2	−0.00030	−0.00089	0.00074	−0.00559	−0.00070	0.00090		
3	0.00013	−0.00075	0.00057	−0.00502	−0.00003	0.00087		
4	−0.00053	−0.00128	−0.00026	−0.00528	−0.00063	0.00024		
5	−0.00069	−0.00197	−0.00041	−0.00570	−0.00080	−0.00056		
6	−0.00002	−0.00200	−0.00058	−0.00628	0.00019	−0.00037		
**Panel B: Cumulative Abnormal Returns (CARs)**								
	**Firms in heavy-polluting industries**		**Firms in non-heavy-polluting industries**					
	Mean1	p-value	Mean2	p-value	Difference	Difference T-test	Two tail p-value	One-tail p-value (Mean1<Mean2)
CAR (−5, −1)	−0.00292	0.052	0.00112	0.262	−0.00405	−2.166 **	0.030	0.015
CAR (−5, 0)	−0.00406	0.016	0.00144	0.193	−0.00550	−2.656 ***	0.008	0.004
CAR (−4, 0)	−0.00509	0.001	0.00156	0.121	−0.00665	−3.503 ***	0.000	0.000
CAR (−3, 0)	−0.00386	0.004	0.00174	0.055	−0.00560	−3.324 ***	0.000	0.000
CAR (−2, 0)	−0.00355	0.003	0.00171	0.025	−0.00526	−3.643 ***	0.000	0.000
CAR (−1, 0)	−0.00252	0.011	0.00170	0.008	−0.00422	−3.527 ***	0.000	0.000
CAR (−5, 1)	−0.00338	0.062	0.00125	0.304	−0.00463	−2.038 **	0.042	0.021
CAR (−5, 2)	−0.00264	0.101	0.00056	0.668	−0.00320	−1.751 *	0.080	0.040
CAR (−5, 3)	−0.00207	0.290	0.00052	0.709	−0.00259	−1.005	0.315	0.157
CAR (−5, 4)	−0.00233	0.274	−0.00010	0.946	−0.00223	−0.801	0.423	0.212
CAR (−5, 5)	−0.00275	0.214	−0.00090	0.562	−0.00184	−0.643	0.520	0.260
CAR (−7, 6)	−0.00628	0.013	−0.00037	0.829	−0.00590	−1.848 *	0.065	0.032

**Notes:** The table presents the average daily abnormal returns (ARRs) and cumulative abnormal returns (CARs) of 2619 listed firms around the passage of the Environmental Protection Tax Law. Total number of the sample was 2619, the subsample for firms in heavy-polluting industries covered 721 observations, while the subsample for firms in non-heavy-polluting industries had 1898 observations. The CARs were calculated using market model regressions and averaged over each event window. ***, **, and * denote statistical significance at the 1, 5, 10% levels, respectively.

**Table 4:** Cross-sectional analysis for firms in heavy-polluting industries.

Variables	CAR (-5, 0)				
	Model 1	Model 2	Model 3	Model 4	Model 5
Fsize	0.002	0.002	0.003 *	0.000	0.001
	(1.133)	(0.892)	(1.819)	(0.141)	(0.570)
Fage	−0.001 **	−0.001 **	−0.001 **	−0.001 ***	−0.001 **
	(−2.484)	(−2.247)	(−2.410)	(−2.708)	(−2.442)
Tobin’s Q	−0.000	−0.000	−0.000	−0.000	−0.000
	(−0.296)	(−0.246)	(−0.256)	(−0.226)	(−0.069)
ROE	−0.018 *	−0.019 **	−0.019 **	−0.017 *	−0.019 **
	(−1.856)	(−1.976)	(−1.980)	(−1.770)	(−1.967)
Dratio	−0.023 **	−0.019 **	−0.021 **	−0.020 **	−0.015
	(−2.320)	(−1.979)	(−2.188)	(−1.978)	(−1.560)
Econcertation	−0.000 **	−0.000 **	−0.000 **	−0.000 **	−0.000 **
	(−2.333)	(−2.404)	(−2.262)	(−2.204)	(−2.168)
Idirector	−0.020	−0.015	−0.026	−0.021	−0.022
	(−0.720)	(−0.529)	(−0.947)	(−0.761)	(−0.800)
SOE	0.005	0.006	0.006	0.003	0.004
	(1.099)	(1.182)	(1.283)	(0.742)	(0.997)
Rlegal		0.001 ***			0.001 ***
		(2.918)			(2.735)
Heavy-pollutingF			−0.016 ***		−0.017 ***
			(−4.227)		(−4.687)
EimprovementA				0.015 ***	0.016 ***
				(4.368)	(4.537)
EimprovementC				0.001	0.004
				(0.276)	(1.089)
Constant	−0.013	−0.012	−0.035	0.021	0.000
	(−0.323)	(−0.291)	(−0.863)	(0.518)	(0.012)
Industry effect	Yes	Yes	Yes	Yes	Yes
Region effect	Yes	Yes	Yes	Yes	Yes
Observations	650	650	650	650	650
R-squared	0.071	0.084	0.099	0.093	0.136
Adjusted R-squared	0.046	0.058	0.073	0.066	0.107
F-value	2.203 ***	2.716 ***	2.792 ***	3.159 ***	4.134 ***
Largest VIF	4.24	5.23	4.24	4.25	5.23
Mean VIF	2.02	2.10	1.98	1.95	1.99

**Notes:** The dependent variable was CAR (-5, 0) in each model. White’s heteroscedasticity t-statistics are given in parentheses. *** p<0.01, ** p<0.05, * p<0.1 based on two-tailed tests.

**Table 5:** The summary of hypotheses and results.

Hypotheses	Results
**Hypothesis 1.** *The market reaction to the passage of Environmental Protection Tax Law is negative for firms in heavy-polluting industries.*	Supported at the 1 % significant level in Table 3
**Hypothesis 2.** *Firms in heavy-polluting industries located in a region with higher-quality legal institutions generates a less negative market reaction around the passage of the Environmental Protection Tax Law.*	Supported at the 1 % significant level in Table 4
**Hypothesis 3.** *Firms in heavy-polluting industries disclosing its heavy-polluting status in annual reports generate a more negative market reaction around the passage of the Environmental Protection Tax Law.*	Supported at the 1 % significant level in Table 4
**Hypothesis 4a.** *Firms in heavy-polluting industries experience a less negative market reaction around the passage of the Environmental Protection Tax Law if they reveal environmental improvement activities using a narrative language.*	Supported at the 1 % significant level in Table 4
**Hypothesis 4b.** *Firms in heavy-polluting industries experience a less negative market reaction around the passage of the Environmental Protection Tax Law if they reveal environmental improvement activities using a quantitative data.*	Partially Supported in Table 4

**Table 6:** Robustness check.

**Panel A: The results of Hausman test for key variables**					
Tested variables	Chi2	Prob>chi2	Results		
Heavy-pollutingF	0.76	1.0000	The variable is exogenous.		
EimprovementA	0.06	1.0000	The variable is exogenous.		
EimprovementC	3.14	1.0000	The variable is exogenous.		
**Panel B: Estimating results using CARs based on different event windows**					
**CAR (−5, −1)**					
**Variables**	**Model 1**	**Model 2**	**Model 3**	**Model 4**	**Model 5**
Rlegal		0.001 ***			0.001 **
		(2.707)			(2.547)
Heavy-pollutingF			−0.014 ***		−0.015 ***
			(−4.001)		(−4.367)
EimprovementA				0.012 ***	0.012 ***
				(3.768)	(3.923)
EimprovementC				0.001	0.003
				(0.206)	(0.987)
Constant	−0.038	−0.036	−0.056	−0.010	−0.028
	(−1.000)	(−0.981)	(−1.511)	(−0.275)	(−0.768)
Industry effect	Yes	Yes	Yes	Yes	Yes
Region effect	Yes	Yes	Yes	Yes	Yes
Observations	650	650	650	650	650
R-squared	0.078	0.089	0.101	0.093	0.130
Adjusted R-squared	0.053	0.063	0.076	0.066	0.101
F-value	1.932 ***	2.564 ***	2.407 ***	2.520 ***	3.498 ***
**CAR (−5, 1)**					
**Variables**	**Model 1**	**Model 2**	**Model 3**	**Model 4**	**Model 5**
Rlegal		0.001 **			0.001 **
		(2.255)			(2.076)
Heavy-pollutingF			−0.017 ***		−0.018 ***
			(−4.150)		(−4.535)
EimprovementA				0.014 ***	0.015 ***
				(3.952)	(4.160)
EimprovementC				0.000	0.003
				(0.073)	(0.778)
Constant	0.073	0.074 *	0.051	0.107 **	0.085 *
	(1.626)	(1.669)	(1.158)	(2.313)	(1.906)
Industry effect	Yes	Yes	Yes	Yes	Yes
Region effect	Yes	Yes	Yes	Yes	Yes
Observations	650	650	650	650	650
R-squared	0.073	0.081	0.099	0.090	0.127
Adjusted R-squared	0.048	0.054	0.074	0.063	0.10
F-value	2.406 ***	2.745 ***	2.920 ***	3.119 ***	3.746 ***
**CAR (−5, 2)**					
**Variables**	**Model 1**	**Model 2**	**Model 3**	**Model 4**	**Model 5**
Rlegal		0.001 **			0.001 **
		(2.165)			(2.112)
Heavy-pollutingF			−0.017 ***		−0.019 ***
			(−4.108)		(−4.524)
EimprovementA				0.014 ***	0.015 ***
				(3.829)	(4.071)
EimprovementC				0.003	0.006
				(0.763)	(1.468)
Constant	0.097 **	0.098 **	0.073	0.129 ***	0.106 **
	(2.016)	(2.061)	(1.562)	(2.632)	(2.237)
Industry effect	Yes	Yes	Yes	Yes	Yes
Region effect	Yes	Yes	Yes	Yes	Yes
Observations	650	650	650	650	650
R-squared	0.076	0.084	0.103	0.092	0.130
Adjusted R-squared	0.052	0.057	0.077	0.065	0.101
F-value	2.778 ***	2.910 ***	3.249 ***	3.328 ***	3.804 ***

**Notes:** The dependent variables were CAR (−5, −1), CAR (−5, 1) and CAR (−5, 2) in related regressions respectively. White’s heteroscedasticity t-statistics are given in parentheses. *** p<0.01, ** p<0.05, * p<0.1 based on two-tailed tests.
